# Examining the prevalence and patterns of multimorbidity in Canadian primary healthcare: a methodologic protocol using a national electronic medical record database

**DOI:** 10.15256/joc.2015.5.61

**Published:** 2015-12-22

**Authors:** Kathryn Nicholson, Amanda L. Terry, Martin Fortin, Tyler Williamson, Michael Bauer, Amardeep Thind

**Affiliations:** ^1^Department of Epidemiology and Biostatistics, Schulich School of Medicine and Dentistry, Centre for Studies in Family Medicine, Western University, Ontario, Canada; ^2^Department of Epidemiology and Biostatistics; and Department of Family Medicine, Interfaculty Program in Public Health, Schulich School of Medicine and Dentistry, Western University, Ontario, Canada; ^3^Department of Family Medicine, Université de Sherbrooke, Québec, Canada; ^4^Department of Community Health Sciences, Cumming School of Medicine, University of Calgary, Alberta, Canada; ^5^Department of Computer Science, Western University, Ontario, Canada

**Keywords:** electronic medical records, electronic records, longitudinal data, methodologic protocol, multimorbidity, multi-morbidity, natural history, primary healthcare, primary care, prevalence

## Abstract

In many developed countries, the burden of disease has shifted from acute to long-term or chronic diseases – producing new and broader challenges for patients, healthcare providers, and healthcare systems. Multimorbidity, the coexistence of two or more chronic diseases within an individual, is recognized as a significant public health and research priority. This protocol aims to examine the prevalence, characteristics, and changing burden of multimorbidity among adult primary healthcare (PHC) patients using electronic medical record (EMR) data. The objectives are two-fold: (1) to measure the point prevalence and clusters of multimorbidity among adult PHC patients; and (2) to examine the natural history and changing burden of multimorbidity over time among adult PHC patients. Data will be derived from the Canadian Primary Care Sentinel Surveillance Network (CPCSSN). The CPCSSN database contains longitudinal, point-of-care data from EMRs across Canada. To identify adult patients with multimorbidity, a list of 20 chronic disease categories (and corresponding ICD-9 codes) will be used. A computational cluster analysis will be conducted using a customized computer program written in JAVA. A Cox proportional hazards analysis will be used to model time-to-event data, while simultaneously adjusting for provider- and patient-level predictors. All analyses will be conducted using STATA SE 13.1. This research is the first of its kind using a pan-Canadian EMR database, which will provide an opportunity to contribute to the international evidence base. Future work should systematically compare international research using similar robust methodologies to determine international and geographical variations in the epidemiology of multimorbidity.

## Introduction

In many developed countries, populations are experiencing a transition, where the burden of illness is shifting from acute to long-term or chronic diseases – producing new and broader challenges for patients, healthcare providers, and healthcare systems [1, 2]. The progressive aging of individuals, improved medical services, and advancing health technologies have led to increased survival among patients with chronic disease. While this is a success of modern medicine, this increased survival has resulted in growing numbers of patients living with multiple chronic diseases and experiencing greater healthcare needs [3–10]. Multimorbidity, the coexistence of multiple chronic diseases within an individual, is now recognized as a significant health system cost and a major public health and research priority [6, 9, 11–15].

Although the prevalence of multimorbidity increases substantially with age, this phenomenon is increasingly being seen in younger populations, as recent studies have found larger absolute numbers of primary healthcare (PHC) populations under the age of 65 years living with multimorbidity [4, 8]. Generally, a PHC population consists of patients seeking integrated and accessible care from a practitioner who: (1) is the first level of contact with the healthcare system; (2) addresses the large majority of personal healthcare needs; and (3) develops a sustained partnership with patients in the context of family and community [16, 17]. Multimorbidity is recognized as the norm, rather than the exception, in PHC populations [10, 18]. In fact, the focus of PHC in many developed countries, including Canada, is principally centered on the treatment and management of chronic diseases, which are often occurring in multiples. Deemed an “endless struggle” by PHC providers, patients experiencing multimorbidity require an integrated healthcare system that adequately responds to their complex and changing needs [19, 20]. These patients represent unique clinical profiles, suffering from distinct combinations of chronic diseases, which can escalate the challenge for providers [21–23]. Clinical and epidemiologic research has yet to provide robust data and evidence on multimorbidity, comparable to information that is readily available for single chronic diseases [24]. Enhanced understanding of multimorbidity prevalence, characteristics, determinants, and prognosis over time is still needed.

Multimorbidity has been conceptualized in many different ways in previous literature, and to date, no “gold standard” measure of multimorbidity has been established. Diederichs *et al*. [25] conducted a systematic review that identified 39 different multimorbidity measures. Some measures are based on simple counts of chronic diseases (with considerable variation in the “list” of diseases used), while other measures differentially weight diseases to account for burden of illness or number of body systems affected [25, 26]. Many commonly used measures of multimorbidity were originally developed and validated among elderly patient populations or hospital-based populations [27]. The marked variation in study methodologies has produced differing prevalence estimates, even among similar PHC populations. In a recent comparison of three studies examining the prevalence of multimorbidity, prevalence levels reported among PHC patients ranged from 34% to 95%, indicating as much as 61% variation in estimates [24]. Not only does this persistent heterogeneity in methodology create incomparable research findings, it also hinders the ability to make informed health system and health policy decisions [11, 24].

To contribute to the growing international evidence base, a national study examining the prevalence and patterns of multimorbidity from the Canadian PHC perspective will be conducted. Although principally used for clinical purposes, electronic medical records (EMRs) can provide rich insight for academic researchers. These clinical data contain longitudinal, patient-level information that present a unique opportunity to examine both the onset and changing burden of multimorbidity over time [3, 6]. The protocol described herein aims to capitalize on this opportunity. This research will examine the burden of multimorbidity among adult PHC patients in Canada, through the use of EMR data.

## Objectives

The objectives of this research are two-fold. Both objectives will contribute to the understanding of multimorbidity in PHC, using a national EMR database. The first objective is to measure the point prevalence and clusters of multimorbidity among adult PHC patients. This objective will aim to understand the overall burden of multimorbidity among adult PHC patients, as well as the most frequently occurring permutations and combinations of chronic disease diagnoses. The second objective is to examine the natural history and changing burden of multimorbidity over time among adult PHC patients. This objective will examine the time-to-event patterns of multiple chronic disease diagnoses, accounting for both provider- and patient-level baseline predictors.

## Methods

### Study design

The key methodologic considerations that should be explicitly described in cross-sectional and retrospective cohort studies examining multimorbidity are defined as the “Methods Crystals for Multimorbidity” by ­Stewart *et al*. [24]. These elements have been notably absent in previously published multimorbidity literature, yet are important to ensure comparable and transparent findings. Following the “Methods Crystals for Multimorbidity” structure, the main study design elements for this research protocol are described more fully in Table 1 [28–30]. While clinical events and encounters with patients are recorded in the EMR prospectively by PHC providers, this research will utilize a retrospective or historic cohort design using existing EMR data. To be included in both objectives, individuals must have at least one in-office encounter date recorded in the EMR and be identified as “adult” patients (at least 18 years of age) as of their first encounter date. Those patients who are under the age of 18 years at their first encounter date or who do not have a detectable in-office encounter recorded in the EMR will be excluded. Those patients who have opted-out of contributing their data to the EMR database will also be excluded from analyses. Ethical approval has been obtained from the Research Ethics Board at Western University (Approval Notice #104705).

### Data source

For both objectives, data will be derived from the Canadian Primary Care Sentinel Surveillance Network (CPCSSN). The CPCSSN database contains longitudinal, point-of-care data from EMRs, which are extracted on a quarterly basis by CPCSSN data managers from participating PHC practices [31, 32]. These data are then de-identified, cleaned, coded, and transformed into a common data format for compilation into the secure CPCSSN database. As of the data extraction period for this research (September 30, 2013), a total of 600,265 de-identified electronic patient records were collected from 475 PHC providers, referred to as “sentinels” by CPCSSN, in 10 regional networks across Canada. The CPCSSN data elements that will be used contain information on practice characteristics (e.g. geographical location); provider characteristics (e.g. provider birth year, provider sex); patient characteristics (e.g. patient birth year, patient sex, first three letters of residential postal code); and in-office encounters (e.g. encounter date, billing diagnosis codes, encounter diagnosis codes). The majority (approximately 95%) of diagnostic codes within the CPCSSN database are recorded using the International Classification of Disease, 9th Revision (ICD-9) system. As such, these codes will be used to identify chronic disease diagnoses.

### Identifying chronic disease diagnoses

Within the CPCSSN EMR data, there are two potential sources of diagnostic codes that are accessible for research purposes. These two sources are the Billing Diagnosis Codes and the Encounter Diagnosis Codes. Both sets of diagnosis codes are recorded using the ICD-9 system, by administrative staff or PHC providers (e.g. nurses, nurses practitioners, medical residents, family physicians), to reflect the patient’s ongoing health status. Each diagnostic code is documented with an associated date (day, month, and year) on which the diagnosis occurred. Initial data exploration indicated variation in where the majority of diagnosis codes were recorded, between these two sources. For example, some practice sites and/or providers primarily use the Billing Diagnosis Codes to record information, while others use the Encounter Diagnosis Codes to do so. Consequently, to capture the maximum amount of data from the patient record, the average number of Billing Diagnosis Codes (total number of billing diagnosis codes divided by the total number of patient encounters) and the average number of Encounter Diagnosis Codes (total number of encounter diagnosis codes divided by the total number of patient encounters) will be calculated on a patient-by-patient basis. The source (Billing Codes or Encounter Codes) with the larger average number of diagnostic codes will be selected for each patient. In addition to using the maximum amount of diagnostic information and avoiding duplicate diagnoses, this approach will also address the variability in diagnostic recording at the patient, provider and practice levels.

### Identifying patients with multimorbidity

To identify adult patients with multimorbidity, we will use a list of 20 chronic disease categories (and corresponding ICD-9 codes) created by a nationally funded (Canadian Institutes of Health Research) research project examining Patient-Centred Innovations for Persons with Multimorbidity (PACE in MM). This ­community-based primary healthcare (CBPHC) project aims to improve the delivery of appropriate, high-quality, and patient-centered interventions to those with multimorbidity [33, 34]. The list was created based on the international literature that examined the burden of multimorbidity among PHC patients, particularly using comprehensive national EMRs [6, 8, 25, 35–40]. The 20 chronic disease diagnoses in the list are particularly relevant in clinical and general populations in Canada. In a separate study, this list will also be validated to ensure it is fully capturing the complex concept of multimorbidity. The complete list of chronic disease categories, as well as corresponding ICD-9 disease codes, are presented in Table 2. In some categories, overlapping ICD-9 codes are presented to ensure that all relevant codes are captured. For example, in the disease category “Thyroid problem”, a range of disease codes, as well as the individual codes, are presented and can be included. The comparison with previously used lists in multimorbidity research is presented in Table 3 [4, 8, 25, 35–48].

### Data analyses

The first objective will examine the overall burden of multimorbidity in terms of its point prevalence and the clusters of multiple chronic disease diagnoses that tend to occur together. For this objective, patients will be followed over time and each chronic disease diagnoses (from the list of 20) received by each patient will be identified. Patient characteristics (e.g. patient age, patient sex, and residential location) will be compared with the broader CPCSSN PHC population, as well as with the general adult Canadian population. Prevalence estimates will be calculated using mutually exclusive count numerators (e.g. patients with 2, 3, 4, and 5 or more chronic diseases) and for each calculation, the denominator will be all eligible adult PHC patients (N=367,743). Prevalence estimates, and corresponding 95% confidence intervals, will be calculated using the *proportion* procedure in STATA SE 13.1 [49]. These estimates will be stratified by patient age and sex categories, as well as provider age and sex categories, to investigate distinct patterns of multimorbidity. Additionally, prevalence estimates will be stratified by the patient’s residential location, which will be determined using the patient’s forward sortation area. More specifically, the second character of the patients’ postal code will determine their residence in a rural (second character is a zero) or urban (second character is a value from one to nine) setting as defined by Canada Post. Among patients with multimorbidity, the frequency of ordered and unordered clusters of chronic disease types will be computed using a customized computer program written in JAVA. The most commonly occurring combinations and permutations of chronic diseases will be presented.

**Figure 1:**
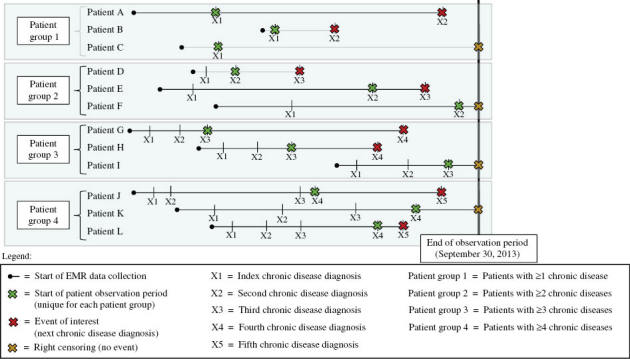
Examples of Objective 2 patient groups, as well as corresponding start and end of observation periods for time-to-event analyses. EMR, electronic medical record.

The second objective will examine the time-to-event patterns of multimorbidity by observing the time elapsing between subsequent chronic disease diagnoses. For this objective, patients with at least one chronic disease diagnosis will be included and four patient groups will be created: (1) patients with one or more chronic disease diagnoses by the end of the observation period; (2) patients with two or more chronic disease diagnoses by the end of the observation period; (3) patients with three or more chronic disease diagnoses by the end of the observation period; and (4) patients with four or more chronic disease diagnoses by the end of the observation period. The details of these patient groups are depicted in Figure 1. The event of interest will be the next chronic disease diagnosis (regardless of diagnosis type). Survival analysis techniques allow for staggered entry dates of patients into the study, as well as right censoring if a patient does not experience the event of interest by the end of the observation period. This will maximize the amount of information contributed by each patient. For all patient groups, the end of the observation period will be September 30, 2013 (date of Q3-2013 extract). A Cox proportional hazards analysis will be used to model time-to-event data, while simultaneously adjusting for provider- and patient-level predictors, and accounting for issues such as patient attrition or delayed entry into observation [50, 51]. The Cox proportional hazards analysis will be conducted using the *stcox* procedure in STATA SE 13.1 [49], and the effects of clustering will be accounted for using a robust variance estimator. Each Cox proportional hazards model will then be built with the provider- and patient-level covariates that report *p*-values of <0.2 in univariate analyses. Interactions among included covariates will be explored, including relevant interaction terms (at a significance level of 0.05) in the final Cox proportional hazards model. The proportional hazards assumption that is inherent in Cox models will be assessed by including time-dependent covariates in the model by using the *tvc* and the *texp* options in the *stcox* procedure. Time-dependent covariates capture interaction of covariates and time. If non-significant, the proportionality assumption is maintained by that covariate. Schoenfeld residuals will also be explored using the *stphtest* procedure, in which the proportionality of the model as a whole and the proportionality for each predictor will be assessed. Once again, non-significant tests indicate no violation of proportionality assumption.

## Discussion

### Anticipated challenges

There are three anticipated challenges of this research: (1) degree of completeness, correctness, and comprehensiveness of the EMR data; (2) limited availability of socioeconomic variables in the EMR data; and (3) the limited generalizability of research findings to the general Canadian population. The first challenge has been well recognized in work that has examined the benefits and limitations of EMR data, particularly for clinical and epidemiologic research [52]. Incomplete or missing data are often a limitation of using EMRs for research, primarily because EMRs are designed to support clinical care delivery and are not structured in a way that easily facilitates use in research [53–55]. Incomplete or free-text data entry by providers may underestimate the prevalence of chronic diseases within the CPCSSN database as these data entries are not included in data extraction or final analysis. This may be particularly true for those diseases with less clear diagnostic features, such as asthma or depression [56, 57]. Before being entered into the final statistical analyses, variables will be assessed for missingness and outliers that may indicate inaccurate data recording.

The second challenge is the lack of availability of sociodemographic variables (e.g. patient ethnicity, education level, employment status, income level) within the Canadian EMRs. When recorded, these variables often contain incomplete data that cannot be used reliably in statistical analyses. This represents an important limitation as previous literature has highlighted the impact of social deprivation (e.g. low income level, low education level, unemployment, barriers to housing) on the development of multimorbidity, particularly at younger ages [3, 4, 8]. Although each patient’s age, sex, and residential location will serve as patient-level predictors of multimorbidity, these variables will not completely account for the socioeconomic factors impacting health. This is indeed an area that requires further attention from providers using EMRs for clinical care.

The third anticipated challenge is that the CPCSSN database does not contain comprehensive data for the entire Canadian population and, therefore, does not represent the burden of multimorbidity for the general adult population in Canada. The CPCSSN database is made up of a selected sample of PHC providers who use EMRs, as well as the patients of these providers. A recent study compared the characteristics of the CPCSSN providers with the respondents of the 2010 National Physician Survey; in which a higher proportion of CPCSSN PHC providers were women and slightly younger in age, while the geographic distribution of the providers was similar to the national characteristics [58]. Likewise, the representativeness of the CPCSSN population was assessed. While this study will compare the characteristics of the adult PHC patients with the characteristics of the broader adult population, in order to determine the degree of generalizability and representativeness of the CPCSSN data, the eventual findings will specifically present the burden of multimorbidity in the PHC setting.

### Anticipated strengths

This research is the first of its kind using a national EMR database, which will provide needed insight and an opportunity to contribute to the international evidence base. Although this clinical information is not principally recorded for research purposes, the CPCSSN database has recently become more accessible to academic researchers for use in innovative projects relevant to CPCSSN’s mission and vision. These data represent the only pan-Canadian EMR database and are recognized as a rich source of PHC information. The previously described approach of identifying chronic disease diagnoses on a patient-by-patient basis will maximize the amount of clinical information derived from each patient’s electronic record, providing insight into PHC beyond what is typically gained from population surveys, administrative databases, and billing information. Furthermore, the computational techniques to determine the most frequently occurring combinations and permutations of multiple chronic diseases will be made accessible to other multimorbidity researchers, with the potential for similar international work.

### Anticipated research outcomes

The first objective will allow for comparisons with international prevalence estimates of multimorbidity and its associated burden; while the second objective will address an important and noted gap in understanding the prognosis of multimorbidity using longitudinal clinical data. The list of 20 chronic diseases for our multimorbidity definition is in accordance with a recent systematic review, which recommended that investigators “should consider the number of diagnoses to be assessed (with at least twelve frequent diagnoses of chronic diseases appearing ideal) and should attempt to report results for differing definitions of multimorbidity (both at least three disease and the classic at least two diseases)” [11]. Finally, this protocol responds to the call for publication of protocols in multimorbidity research and aims to support the transparency, reproducibility, and replication of this research methodology [59]. This could facilitate the creation of comparable estimates of multimorbidity across patient populations, both in ­Canada and abroad.

### Anticipated clinical- and policy-level impact

This research will have both clinical and policy relevance. The complexities of multimorbidity create heterogeneity in the experiences of patients as they cope with and receive clinical management for their multiple chronic diseases. This is further complicated by the heterogeneity in the clinical profile, or disease combination, each patient experiences. Combined with the current lack of evidence-based clinical practice guidelines that facilitate patient-centered and coordinated care, these complex clinical pathways and clinical profiles have significant implications for health-related outcomes and use of healthcare resources. As such, national multimorbidity estimates will help to inform where the redevelopment of clinical practice guidelines must focus to have the greatest clinical impact. From a public health or health policy perspective, the growing burden of multimorbidity consumes considerable societal and economic resources, and negatively impacts satisfaction with care delivery, quality of life, and productivity of patients and their caregivers. Examining the most frequently occurring clusters of chronic disease, and patients who are most at risk of subsequent chronic disease diagnoses, can help inform the development of clinical- or population-level interventions to relieve this tsunami of health demands and to provide robust support needed by all stakeholders [12, 18, 24, 60].

## Conclusion

This protocol aims to examine the prevalence and changing burden of multimorbidity among adult PHC patients using EMR data. As electronic records are increasingly being used for academic research and health system planning, these data must be managed and analyzed properly. The findings of this research will be disseminated through publication and presentation to academic researchers, decision-makers, and healthcare professionals. Future work should systematically compare international research using similar methodologies (e.g. definitions of multimorbidity, data sources, populations of interest) to explore international and geographical variations in the epidemiology of multimorbidity. Finally, a concerted and multifaceted effort must be made to establish effective and patient-­centered interventions that help to alleviate the burden of multi­morbidity for patients, caregivers, and healthcare providers into the future.

## Figures and Tables

**Table 1:** Key methodologic elements of research protocol.

Major category		Methodologic considerations	Details	Methodologic elements	
1.	Design	Research design	–	●	Retrospective or historic cohort study
2.	Population and sampling	Location	–	●	Ten practice-based research networks across Canada; located in eight Canadian provinces: British Columbia, Alberta (two networks), Manitoba, Ontario (three networks), Quebec, Nova Scotia and Newfoundland and Labrador
		Sampling	Sampling method	●	Recruitment of PHC networks, sites, and providers; collection of patient data from the participating PHC providers
		PHC setting	Sampling frame	●	Consenting PHC networks, sites, and providers
			Sampling method	●	Non-random recruitment; based on interest in participating in CPCSSN dataset
			Sampling size	●	82 PHC sites from 10 PHC networks
		PHC providers	Sampling frame	●	39,392 family physicians [28] and 3,655 nurse practitioners in Canada in 2013 [29]
				●	Approximate total of 43,047 PHC providers in Canada in 2013
			Sampling method	●	Non-random recruitment; based on interest in participating in CPCSSN dataset
			Sampling size	●	477 PHC providers recruited and contributing data
		PHC patients	Sampling frame	●	35,345,000 population (and therefore potential PHC patients) in Canada in 2013 [30]
			Sampling method	●	Non-random collection of data; consistent and consecutive data collection
			Sampling size	●	600,565 PHC patients included and contributing data
		Rationale for sample size	–	●	Utilized all eligible, adult patients included in the CPCSSN database
3.	Data and definition	Data collection	Source of data	●	Entire electronic medical record; provider documentation at each encounter
			Method of data collection	●	Extraction of de-identified and anonymized EMR data
		Coding	Morbidity coding	●	ICD-9
		Time	Time period of data source	●	Length of EMR and ending on September 30, 2013 (date of data extract)
			Length of recruitment period for patients for each PHC provider	●	Recruitment began in 2008 and ended as of September 30, 2013 (date of data extract); length of retrospective data entered into the EMR system for each patient varied by PHC providers
			Dates of data collection	●	All data were extracted as of September 30, 2013
			Morbidity time focus	●	Ever diagnosed in the EMR (lifetime morbidity)
		Definitions	Definition of multimorbidity	●	Two or more chronic disease diagnoses; creation of mutually exclusive categories of multimorbidity (patients with 2, 3, 4 and 5+ chronic disease diagnoses)
			Definition of chronic diseases	●	Chronic diseases were identified according to WHO definition of chronic and based on burden among populations; list of 20 chronic disease categories and corresponding ICD-9 codes are included in Table 2
			Operational definition of the count of chronic diseases	●	PHC providers recorded diagnostic code during clinical encounter (not for research purposes); double counting was avoided by identifying the first chronic disease diagnoses of those chronic diseases on list
	4. Outcomes	Results	Outcomes reported	●	Prevalence of single morbidity; prevalence of multiple morbidities; prevalence estimates stratified by relevant covariates; commonly occurring clusters of multiple chronic diseases; time-until-event information on both onset and advancing multimorbidity
			Confounders controlled	●	Site characteristics (geographical location); provider characteristics (age, sex); patient characteristics (age, sex, residential geographic location)
			Results presented	●	Prevalence of multimorbidity; characteristics of adult patients living with multimorbidity; prognosis of multimorbidity over time

CPCSSN, Canadian Primary Care Sentinel Surveillance Network; EMR, electronic medical record; ICD-9, International Classification of Disease, 9th Revision; PHC, primary healthcare; WHO, World Health Organization.

**Table 2:** List of 20 chronic disease categories, and corresponding International Classification of Disease, 9th Revision (ICD-9) disease codes, for identifying adult primary healthcare patients with multimorbidity.*

Chronic disease category		ICD-9 codes
1.	Hypertension	401–405, 401, 401.1, 401.9, 405, 405.01, 405.09, 405.1, 405.11, 405.19, 405.9, 405.91, 405.99
2.	Obesity	278, 278.01
3.	Diabetes	250, 250.01, 250.02, 250.03, 250.1, 250.11, 250.12, 250.13, 250.2, 250.21, 250.22, 250.23, 250.3, 250.31, 250.32, 250.33, 250.4, 250.41, 250.42, 250.43, 250.5, 250.51, 250.52, 250.53, 250.6, 250.61, 250.62, 250.63, 250.7, 250.71, 250.72, 250.73, 250.8, 250.81, 250.82, 250.83, 250.9, 250.91, 250.92, 250.93
4.	Chronic bronchitis, Chronic obstructive pulmonary disease or Asthma	491, 491.1, 491.2, 491.21, 491.22, 491.8, 491.9, 492, 492.8, 493, 493.01, 493.02, 493.1, 493.11, 493.12, 493.2, 493.21, 493.22, 493.8, 493.81, 493.82, 493.9, 493.91, 493.92, 496
5.	Hyperlipidemia	272, 272.1, 272.2, 272.3, 272.4
6.	Cancer	140–239, 140–149, 150–159, 160–165, 170–176, 179–189, 190–199, 200–209
7.	Cardiovascular disease	412, 413, 413.1, 413.2, 440–449, 427, 427.3, 427.31, 417.32
8.	Heart failure	428, 394, 394.1, 394.2, 395, 395.1, 395.2, 395.9
9.	Anxiety or Depression	296, 296.2, 296.21, 296.22, 296.23, 296.24, 296.25, 296.26, 296.3, 296.31, 296.32, 296.33, 296.34, 296.35, 296.36, 300, 300.01, 300.02, 300.09
10.	Osteoarthritis or Rheumatoid arthritis	714, 714.1, 714.2, 714.3, 715, 715.1, 715.2, 715.3, 715.8, 715.9
11.	Stroke or Transient ischemic attack	434, 434.01, 434.1, 434.11, 433.9, 434.9, 434.91, 435, 435.1, 435.2, 435.3, 435.8, 435.9
12.	Thyroid problem	240–246, 240, 241, 242, 243, 244, 245, 246
13.	Chronic kidney disease or failure	585, 585.1, 585.2, 585.3, 585.4, 585.5, 585.6, 585.9
14.	Osteoporosis	733, 733.01, 733.02, 733.03, 733.09
15.	Dementia	290, 290.1, 290.11, 290.12, 290.13, 290.2, 290.21, 290.3, 290.4, 294, 294.1, 294.2
16.	Chronic musculoskeletal problem	723, 723.1, 724, 724.1, 724.2, 724.3, 724.4, 724.5, 725, 726, 726.1, 726.2, 726.3, 726.31, 726.32, 726.33, 726.39, 726.4, 726.5, 726.6, 726.61, 726.62, 726.63, 726.64, 726.65, 726.69, 726.7, 726.71, 726.72, 726.73, 726.79, 726.9, 726.91, 727, 727.01, 727.03, 727.04, 727.05, 727.06, 727.09, 727.2, 727.3, 729, 729.1, 729.2, 729.4, 729.5
17.	Stomach problem	530, 530.81, 531, 531.4, 531.41, 531.5, 531.51, 531.6, 531.61, 531.7, 531.71, 531.9, 531.91
18.	Colon problem	555, 555.1, 555.2, 555.9, 556, 556.4, 556.5, 556.6, 556.8, 556.9, 564, 564.1
19.	Chronic liver disease	571, 571.1, 571.2, 571.3, 571.4, 571.41, 571.42, 571.49, 571.5, 571.6, 571.8, 571.9
20.	Chronic urinary problem	593, 593.3, 593.4, 593.5, 593.7, 593.71, 593.72, 593.73, 593.8, 593.82, 593.89, 593.9, 595, 595.1, 595.2, 595.9, 597, 597.8, 597.81, 597.82, 600, 601, 601.1, 601.3, 601.8, 601.9, 602, 602.1, 602.2, 602.3, 602.8, 602.9

*List reproduced with the permission of co-author and co-principal investigator of the Patient-Centred Innovations for Persons with Multimorbidity (PACE in MM) Community Based Primary Health Care (CBPHC) Team (M.F.).

**Table 3:**
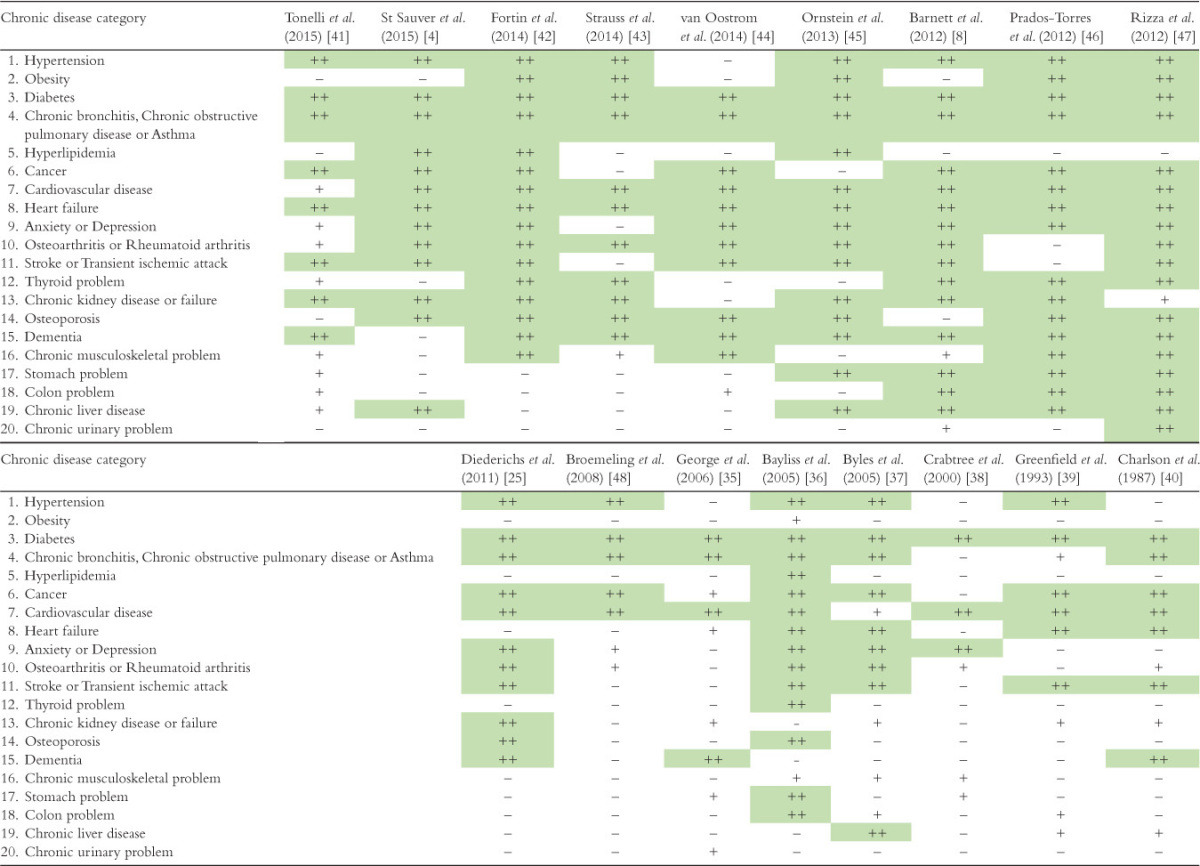
Comparison of multimorbidity/chronic disease lists from publications in the multimorbidity literature, and the current list of 20 chronic disease categories, where “++” indicates comparable disease categories.
